# Full-Scale Model Tests of Two Box-Type Soil–Steel Structures with Different Crown and Haunch Radii

**DOI:** 10.3390/ma17081710

**Published:** 2024-04-09

**Authors:** Fei Wu, Baodong Liu, Weiming Sun, Haibo Sun, Shun Zhang

**Affiliations:** 1 School of Civil Engineering, Beijing Jiaotong University, Beijing 100044, China; 20115012@bjtu.edu.cn (F.W.); 19121104@bjtu.edu.cn (W.S.); 2 Shandong Provincial Transportation Planning and Design Institute Company Ltd., Jinan 250031, China; shbjinan@163.com (H.S.); 18121165@bjtu.edu.cn (S.Z.)

**Keywords:** box-type soil–steel structures, full-scale tests, cross-section, mechanical performance, modified calculated method

## Abstract

Compared with circular, arched, and pipe-arched soil–steel structures, box-type soil–steel structures (BTSSSs) have the advantages of high cross-section utilization and low cover depth. However, the degree of influence of the crown and haunch radii on the mechanical performance of BTSSSs is still unclear. Therefore, two full-scale BTSSS models with a span of 6.6 m and a rise of 3.7 m but with different crown and haunch radii were established, and the mechanical properties during backfilling and under live load were tested. Afterward, 2D finite element models (FEMs) were established using the ABAQUS 2020 software and verified using the test data. The influence of cross-section geometric parameters on mechanical performance was analyzed by using the FEM, and a more accurate formula for calculating the bending moment during backfilling was proposed. The results show that the BTSSS with a smaller crown radius has a stronger soil–steel interaction, which promotes more uniform stress on the structure and makes the structure have smaller relative deformations, bending moments, and earth pressure. The span and arch height greatly influence the bending moment and deformation of the structure. Based on the CHBDC, the crown and haunch radii were included in the revised calculation formula.

## 1. Introduction

Soil–steel structures are widely used nowadays as an efficient solution for roadways overpasses, culverts, and bridges [1,2]. They offer cost-effective and easy-to-construct alternatives to conventional concrete and steel bridges [3]. However, the cross-section utilization rate of the traditional circular, arched, and pipe-arched soil–steel structures is low. To meet the clearance requirements, the span and rise of the structures often need to be increased, which is not suitable for roads with low embankments in plain areas. The box-shaped cross-section of the box-type soil–steel structures (BTSSSs) makes the BTSSS have a higher cross-section utilization rate compared with the arched soil–steel structures [4]. At the same time, the minimum cover depth of BTSSSs specified in the specification [5,6] is small. Therefore, they are widely used in urban underpasses, plain area railways, and highways.

Scholars have conducted extensive research on BTSSSs through field tests, full-scale model tests and numerical analysis. Duncan et al. [7] conducted a loading test on an aluminum BTSSS with a span of 5.3 m and a rise of 1.9 m and analyzed the effects of the span, rise, cover depth, vehicle load, load position, degree of compaction on the bending moment, and axial force of BTSSS through finite element models (FEMs). The research results show that the influence of the axial force is very small compared with the bending moment, and only the bending moment can be considered in the design. Duncan fitted the calculation formula for crown and haunch bending moments with span and cover depth as variables. This approach was adopted by American Association of State Highway and Transportation Officials (AASHTO) LRFD Bridge Design Specifications [5], CSA Canadian Highway Bridge Design Code (CHBDC) [6], and Australian/New Zealand Standard (AS/NZS) [8]. Byrne [9] tested the response of a 4.8 m span and 1.5 m rise BTSSS with stiffeners during backfilling and under live load. The research results showed that the stiffeners effectively reduced the deformation of the structure. The shallow cover and box-shaped geometry influence negative arching. Flener et al. [10,11,12] tested the response of two full-scale BTSSS models with spans of 8 and 14 m during the backfill process, under vehicle load, and in the ultimate state and compared the test results with the Swedish design method (SDM) [13] and CHBDC [6]. The test results show that the span has a great influence on the response of the structure during the construction and loading process, and the strengthening of the crown can significantly reduce the deformation and stress of the structure. As the cover depth increases, the effect of strengthening decreases gradually, and the bearing capacity increases linearly. McCavour et al. [14] conducted loading tests on two 12 m span BTSSS models stiffened with different methods and conducted numerical analysis through NLSSIP to study the effects of stiffening methods and degree of compaction on structural performance. Korusiewicz and Wysokowski [15,16,17] tested the mechanical response of a BTSSS model with a span of 3.55 m and a height of 1.42 m during backfilling and loading and analyzed the impact of a single layer of geotextile on the load-bearing capacity of the structure. The research results show that the application of a geotextile in the backfill layer reduces the displacements by up to 30%. The bearing capacity and stiffness are also significantly improved.

In conclusion, the mechanical performance of BTSSSs has been extensively studied. The cover depth of the BTSSS is usually less than 1.5 m. When the clearance and elevation of pavement inside and above the structure are determined, the cover depth decreases as the crown radius decreases. It is obvious that a smaller crown radius facilitates the soil–steel interaction, while an increase in cover depth facilitates the spread of live loads [18,19]. The coordination between the above two factors requires designers to pay attention. The influence of cover depth has been extensively studied, while the influence of crown radius on structural performance has been less studied [20,21]. The above factors are not considered in the calculation method proposed by Duncan. Pettersson [22] added a coefficient considering the ratio of the crown radius to the haunch radius to the formula proposed by Duncan and compared the calculated results with the test results of Flener’s full-scale model. When the crown radius is infinite, the internal force calculation formula proposed by Pettersson is infinite. This is evidently inconsistent with objective facts. The formula proposed by Pettersson was adopted by the SDM [13]. BTSSSs also have a broader application prospect in box culvert and slab culvert extension projects. To match the cross-section of the existing culvert structure, the crown radius of the newly added BTSSSs is usually large. This usually does not meet the applicable scope of the formulas in AASHTO and AS/ZNS, so its internal forces need to be calculated by complex FEMs. Insufficient research and flaws in design methods lead to difficulties for designers in selecting the cross-section of BTSSSs.

For the purpose of studying the influence of crown and haunch radii on the response of the BTSSS during backfilling and under live loads, two full-scale models with different crown and haunch radii were established. The span and rise of the two full-scale models were 6.6 and 3.7 m, respectively. Subsequently, reliable FEMs were developed using ABAQUS, and parametric analyses were conducted to evaluate the effects of geometric parameters on the behavior of the BTSSS. Finally, the calculation formula for the internal force of the BTSSS in the backfilling process considering the crown and haunch radii was proposed.

## 2. Experimental Scheme

### 2.1. Description of the Full-Scale Model

**Figure 1:**
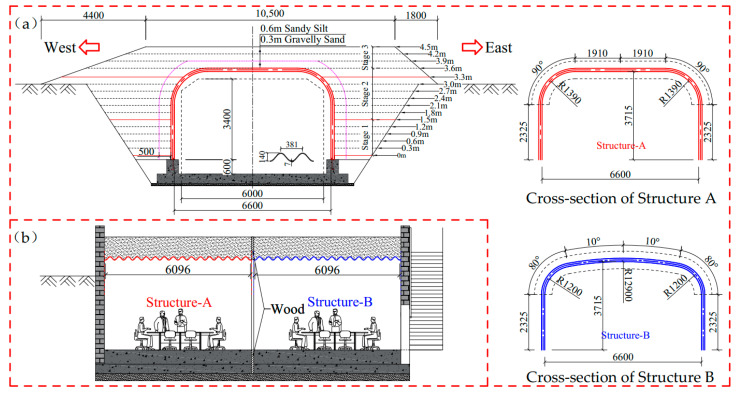
Full-scale model of BTSSS: (**a**) cross-section; (**b**) longitudinal section (dimensions in (mm)).

The design of the two full-scale models refers to the clearance requirements for bridges over rural roads in the JTG D60-2015 [23]. The horizontal and vertical clearance of the two full-scale models are 6 and 4 m, respectively. The span, rise, and sidewall height of the two full-scale models are 6.6, 3.7, and 2.33 m, respectively, as shown in Figure 1a. The difference between the two full-scale models is the radius of the crown and haunch. One of the models has an infinite crown radius (the crown of the structure is flat) and a haunch radius of 1.4 m, which will be called Structure A. The other model has a crown radius of 12.9 m and a haunch radius of 1.2 m, which will be called Structure B. The two full-scale models are made of deep corrugated steel plates (CSP) with 381 mm pitch, 140 mm depth, and 6 mm thickness. The yield strength and elasticity modulus of the CSP are 355 MPa and 206 GPa. The length of both models is 6.1 m. The CSP, foundation, and backfill of the two models are separated from each other. The gaps between the CSP of the two models are covered with thin steel plates, and the backfills are separated wooden boards, as shown in Figure 1b. The two models were backfilled synchronously. Gravelly sand is used for backfilling within 50 cm on both sides of the sidewall and 0.3 m above the crown, and the remaining areas are backfilled with sandy silt. Backfill around the structures is placed in lifts of 30 cm thick layers and then compacted to 95–100% optimum density according to standard proctor test in compliance with the construction requirements of AASHTO [5] and the CHBDC [6].

### 2.2. Test Program and Experimental Procedure

**Figure 2:**
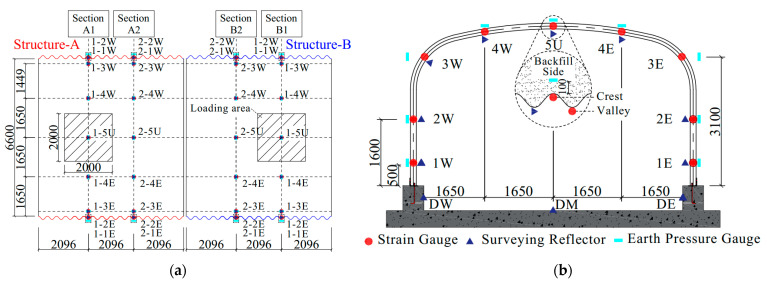
Surveying points: (**a**) top view; (**b**) transverse section (dimensions in (mm)).

**Figure 3:**
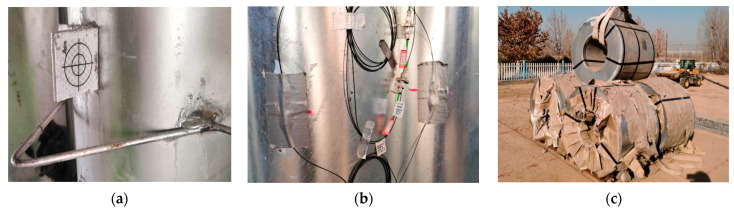
Test and loading device: (**a**) surveying reflector; (**b**) FBG strain sensors; (**c**) loading device.

The performance of the structure was monitored by surveying points at two transverse sections within each structure located at the third point of the bridge length (Figure 2a). Section A1 and B1 were the main test sections, and section A2 and B2 were the control sections. The displacement and strain measurement points of section A/B-1 were arranged at the crown, haunch, 1/4 of the span, and the position of 1.6 and 0.5 m above the foot of the sidewall, as shown in Figure 2b. The control sections A2 and B2 lacked unimportant measuring points 4E and 1E compared to the main test sections A1 and B1. In addition, the displacement of the foundation was measured through the measuring points on the middle and side walls of the foundation. The displacement of the structure was measured by using a Keda Total Station 442R10, Guangdong Southern Era Technology Co., Ltd., Guangzhou, China which provides a distance accuracy of 1 mm. The surveying reflectors were attached to the valley of the CSP and the surface of the foundation, as shown in Figure 3a.

The strain in the circumferential direction of the CSP was measured using 64 fiber Bragg grating (FBG) strain sensors, Shenzhen Zhongke Sensing Technology Co., Ltd., Shenzhen, China installed over the rings. A pair of FBG strain sensors were installed at the crest and valley of each measuring point, as shown in Figure 3b. The axial force and bending moment were calculated from strains based on the plane cross-section assumption. The wavelength of the FBG strain sensors is affected by strain and temperature at the same time [24], so the temperature change near the measurement points was measured using thermocouple probe thermometers. The vertical earth pressure of the crown and the horizontal earth pressure of the sidewall and haunch of the models were also measured using FS-TY-04 single-membrane vibrating wire earth pressure cells, Jiangxi Feishang Technology Co., Ltd., Nanchang, China. The position of the earth pressure cells corresponded to the displacement and strain measurement points. The earth pressure cells were arranged at a distance of 10 cm from the CSP to reduce the interference of the uneven surface on the test results.

The displacement, strain, and earth pressure of the two models during backfilling were monitored after each backfill layer was placed on both sides of the CSP. When backfilling to 30, 60, and 90 cm above the crown, the loading tests were performed on Structures A and B using the steel strip coils, as shown in Figure 3c. The loading weights were determined to be 170, 240, and 280 kN, according to the axle weight of the design truck and the load factor in JTG D60-2015 [23]. Moreover, 170 kN is the rear axle load 140 kN of the design truck multiplied by the load factor 1.2. Furthermore, 240 kN and 280 kN are respectively twice the middle and rear axle load of the design trucks. A square pallet with a width of 2 m was set under the coils.

## 3. Mechanical Behavior of BTSSS during Backfilling

### 3.1. Structure Deformation during Backfilling

This paper stipulates that eastward horizontal deformations and upward vertical deformations are positive. According to the test results, the horizontal deformation of the sidewall during backfilling is relatively large, and the horizontal deformation of 2W and 2E is the most significant. The vertical deformation of the crown and one-quarter span is relatively large, but the horizontal deformation is small. Therefore, the horizontal deformation of measuring points 2W and 2E and the vertical deformation of measuring points 4W and 5U during backfilling are analyzed below.

**Figure 4:**
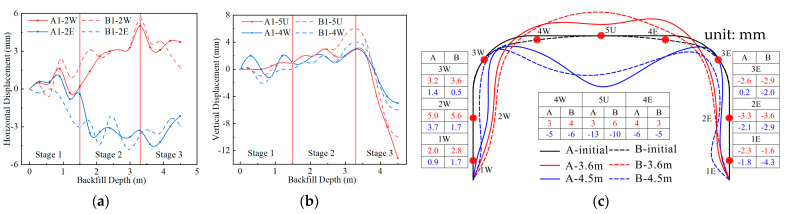
The deformation of Structures A and B during backfilling: (**a**) horizontal deformation of 2W and 2E; (**b**) vertical deformation of 4W and 5U; (**c**) deformation at the end of stages 2 and 3 (scaled up 100 times).

Figure 4a shows the horizontal deformation of measuring points 2W and 2E during the backfilling process. Figure 4b shows the vertical deformation of measuring points 4W and 5U. The deformation shown in Figure 4 has been subtracted from the foundation settlement. According to the deformation law of the structure during backfilling, the backfilling process is divided into three stages. Stage 1 is backfilling to 1.5 m above the arch foot (backfilling to measuring points 2W and 2E); stage 2 is backfilling to 3.3 m above the arch foot (backfilling to the haunch); and stage 3 is backfilling to 90 cm above the crown, as shown in Figure 1a. Figure 4c shows the overall deformation of Structures A and B at the end of stages 2 and 3.

It can be seen from Figure 4 that due to the loading and unloading during the compaction process, the deformation of the measuring point fluctuates. The inward deformation of the sidewall and the upward deformation of the crown increase in stages 1 and 2. Compared with the deformation in stage 1, the deformation in stage 2 is more obvious. Due to the greater radius of the crown of Structure A, the horizontal restraint on the sidewall is stronger, so the deformation of Structure A in stages 1 and 2 is smaller than that of Structure B. The upward deformations of Structures A and B at the end of stage 2 are 3 and 6 mm, respectively. After backfilling to the haunch (stage 3), the overlying soil above the haunch exerts vertical earth pressure on the structure. The crown of the structure begins to deform downward, and the sidewall begins to deform outward. At the end of backfilling, the vertical deformations of Structures A and B are 13 and 10 mm, respectively, and the deformation of Structure A is 1.3 times that of Structure B.

### 3.2. Stress and Internal Force during Backfilling

**Figure 5:**
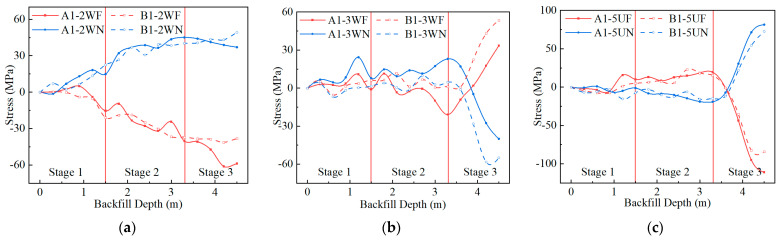
The stresses of Structures A and B during backfilling: (**a**) 2W; (**b**) 3W; (**c**) 5U.

**Figure 6:**
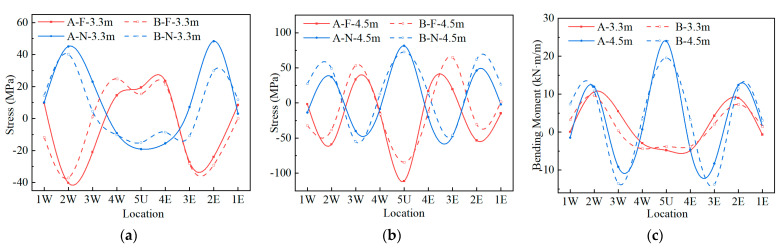
The stresses and bending moments of Structures A and B during backfilling: (**a**) stress when backfilling to 3.3 m; (**b**) stress when backfilling to 4.5 m; (**c**) bending moment when backfilling to 3.3 and 4.5 m.

This paper stipulates that the stress in tension and the bending moment causing the inner side of the CSP to be in tension are positive. During the backfilling process, the stress changes in measuring points in the middle of the sidewall, haunch, and crown are relatively significant. Therefore, this paper analyzes the stress change process of 2W, 3W, and 5U during backfilling. The stress changes in the above measuring points during backfilling are shown in Figure 5. The BTSSS is mainly able to bear the bending moment [7], and the tested stress is converted into the bending moment according to the plane cross-section assumption and the generalized Hooke’s law. The stresses and moment distributions of the two structures at the end of stages 2 and 3 are shown in Figure 6. The letters ‘F’ and ‘N’ in the legends of Figure 5 and Figure 6 represent the stress at the crest and valley positions of CSP, respectively. The relative positions of the crest and valley are shown in Figure 2b. According to Figure 5 and Figure 6, the inner side of the sidewall and haunch and the outer side of the crown of CSP are in tension in stages 1 and 2. At the end of stage 2, the tensile stress at measuring point 2W is the largest. The maximum tensile stresses of Structures A and B are 48.4 and 40.2 MPa, respectively.

In stage 3, under the influence of the vertical earth pressure of the crown, the stresses at the haunch and crown measuring points begin to decrease and gradually reverse, while the stress at the sidewall does not change significantly. At the end of stage 3, there is tension on the inside of the crown and sidewall measurement points and the outside of the haunch measurement point. Both Structures A and B have the largest compressive stress on the outside of the crown at the end of stage 3. The maximum stresses of Structures A and B are 111 and 84 MPa, respectively. However, due to the difference in stress at the end of stage 2 or mechanical performance, the stress at the haunch of Structure A is smaller than that of Structure B.

### 3.3. Earth Pressure during Backfilling

**Figure 7:**
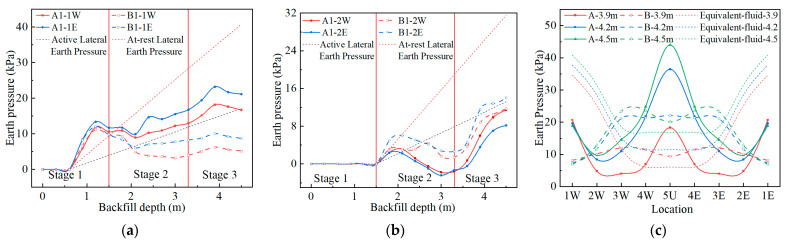
The earth pressure of Structures A and B during backfilling: (**a**) 1W, 1E; (**b**) 2W, 2E; (**c**) earth pressure distribution.

During the backfilling process, the horizontal earth pressure of the sidewall and haunch and the vertical earth pressure of the crown were measured using the earth pressure cell. There are many test conditions for measuring points 1W, 1E, 2W, and 2E, so the change in horizontal earth pressure of the above measuring points during the whole backfilling process was analyzed, as shown in Figure 7a,b. Figure 7c shows the earth pressure distribution after the backfill reaches the haunch. The measured vertical and horizontal earth pressures were respectively compared with the lateral earth pressure calculated by the equivalent fluid method, with the at-rest lateral earth pressure and active lateral earth pressure calculated according to the AASHTO [5]. When the earth pressure was calculated, the bulk density and friction angles of sandy silt and gravelly sand were taken from the results of compaction and triaxial compression tests.

It can be seen from Figure 7a,b that the horizontal earth pressures of 1W, 1E, 2W, and 2E under the influence of compaction are greater than the at-rest lateral earth pressures in the first two layers after the earth pressure cell was buried. With the reduction in compaction influence and the inward deformation of the sidewall, the earth pressure of the sidewall decreases in the following three conditions and is gradually smaller than the active lateral earth pressure. After that, the horizontal earth pressures of 1W and 1E increase with the increase in fill height. Since the displacements of 1W, 1E, 2W, and 2E begin to reversely change after the backfill reaches the haunch, the earth pressures of 2W and 2E increase significantly but are always lower than the at-rest lateral earth pressure.

It can be seen from Figure 7c that under the influence of non-dissipated compaction force or the negative soil arch formed by the high stiffness of the structure, the vertical earth pressure of 5U, 4W, and 4E of both structures is greater than that calculated by the equivalent fluid method after backfilling to the crown. During the backfilling process, the earth pressures of 1W and 1E of Structure A are greater than that of Structure B, while the earth pressure of 2W, 2E, 3W, and 3E is lower than that of Structure B. The vertical earth pressure of the crown of Structure A is significantly greater than that of the one-quarter span, while the difference in vertical earth pressure between the crown and one-quarter span of Structure B is small. The crown earth pressure of Structure A is significantly greater than that of Structure B, and the crown earth pressure of Structure A at the end of backfilling is 2.2 times that of Structure B.

## 4. Mechanical Behavior of BTSSS under Live Loading

### 4.1. Structure Deformation under Live Loading

**Figure 8:**
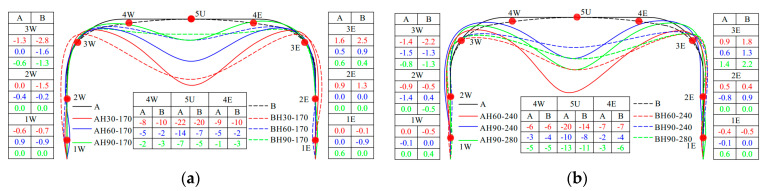
The deformation of Structures A and B under live load (magnification 80 times): (**a**) 170 kN; (**b**) 240, 280 kN. (Unit: mm).

Figure 8a shows the deformation of the structure under a 170 kN load. Figure 8b shows the deformation of the structure under 240 and 280 kN loads. It can be seen from Figure 8 that the vertical deformation of the crown of Structure A is greater than that of Structure B under the same load and cover depth and is 1.1 to 2 times that of Structure B. The crown deformation of Structure B under live load is relatively gentle. Under the same load, the haunch horizontal deformation of Structure A is smaller than that of Structure B, and the soil–steel interaction of Structure B is more intense. It can be seen from Figure 8a that the vertical deformation under the same load decreases with the increase in cover depth. The live load diffusion area increases with the increase in cover depth. This limits the transformation of the structure from vertical deformation to horizontal deformation, and the difference in horizontal deformation decreases as cover depth increases.

### 4.2. Internal Force under Live Loading

**Figure 9:**
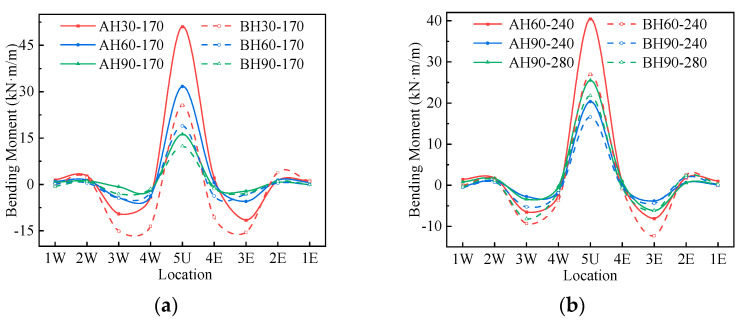
The bending moments of Structures A and B under live load: (**a**) 170 kN; (**b**) 240, 280 kN.

The bending moments of Structures A and B under the live load are shown in Figure 9. It can be seen from Figure 9 that the bending moment distribution corresponds to the deformation under the live load shown in Figure 8. The inner side of the crown and the outer side of the haunch are under tension, and the crown and haunch bending moments are larger. There are two inflection points between measuring points 4 and 5 and between measuring points 3 and 2. Because the soil–steel interaction of Structure B is stronger, the crown bending moment of Structure B is more transferred into the haunch bending moment under the live load. Therefore, the crown bending moment of Structure A is greater than that of Structure B under the same loading conditions, and the haunch bending moment of Structure A is less than that of Structure B. When the cover depth is 30 cm and the load is 170 kN, the crown bending moment of Structure A is twice that of Structure B. The difference between the crown and haunch bending moments of Structure A and that of Structure B decreases gradually with the increase in cover depth.

**Figure 10:**
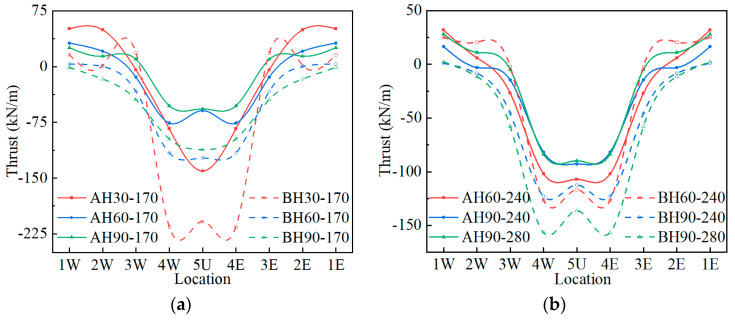
The thrust of Structures A and B under live load: (**a**) 170 kN; (**b**) 240, 280 kN.

The axial forces of Structures A and B under the live load are shown in Figure 10. The negative values in Figure 10 represent the compressive axial force. It can be seen from Figure 10 that the crown and one-quarter span measuring points bear the compressive axial force under the live load. The compressive axial force of Structure B is significantly greater than that of Structure A under the same load conditions. When the cover depth is 30 cm and the load is 170 kN, the crown axial force of Structure B is 1.48 times that of Structure A. This prompts Structure B to exert stronger soil–steel interaction. As the cover depth increases, the difference in axial force decreases, and so does the difference in the bending moment of the structure. As the cover depth increases, the tensile axial force of the structure also gradually decreases.

### 4.3. Earth Pressure under Live Loading

**Figure 11:**
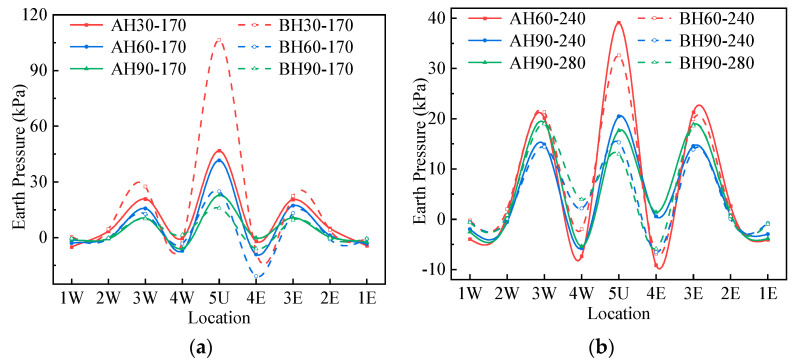
The earth pressure of Structures A and B under live load: (**a**) 170 kN; (**b**) 240, 280 kN.

Figure 11 shows the additional earth pressure distribution of the structure under the live load. It can be seen from Figure 11 that the vertical earth pressure of the crown and horizontal earth pressure of the haunch change greatly. The horizontal earth pressure at measuring points 1 and 2 and the vertical earth pressure at measuring point 4 have small changes and appear to decrease under some loading conditions.

Since the deformation of the crown of Structure B is relatively average and small, the vertical earth pressure of the 5U of Structure B is smaller than that of Structure B, and the earth pressure of the one-quarter span position is greater than that of Structure B when the cover depth is 60 and 90 cm. Since the roof of Structure B is arc-shaped, the pressure at the crown is relatively concentrated when the cover depth is small. Therefore, when the cover depth is 30 cm, the crown vertical earth pressure of Structure B is greater than that of Structure A. Since the horizontal deformation of Structure B is more obvious than that of Structure A when the cover depth is 30 cm, the haunch horizontal earth pressure of Structure B is greater than that of Structure B. When the cover depth is 60 and 90 cm, the difference in horizontal deformation between Structures A and B is small, so the haunch horizontal earth pressure is not much different. It is worth noting that the haunch horizontal earth pressure by loading 280 kN is greater than that of 240 kN when the covering soil is 90 cm, while the crown vertical earth pressure is the opposite. This shows that both Structures A and B exhibit strong soil–steel interaction. When the load increases, the earth pressure at the crown shifts to both sides, which results in the release of the earth pressure at the crown.

## 5. Numerical Simulation and Results

### 5.1. Numerical Modeling

**Figure 12:**
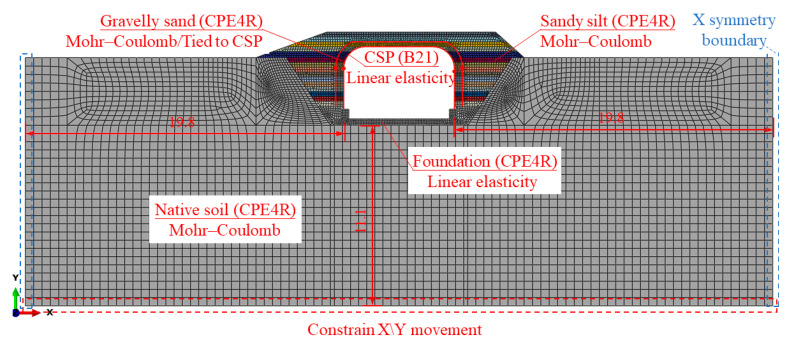
FEM built in ABAQUS (dimensions in (m)).

For improving computational efficiency, 2D FEMs (as shown in Figure 12) were established by the ABAQUS 2020 software to obtain the performance of the BTSSS during the backfilling process and under the live load [25,26,27]. The native soil was extended to three times the span of the BTSSS, and symmetric boundary conditions (Ux = URy = URz = 0) were imposed [28]. The distance from the bottom boundary of the native soil to the foundation was taken as 11.1 m, about three times the rise of the BTSSS, and all degrees of freedom of the nodes on the bottom boundary were fixed. The two-node liner beam elements (B21) were used to simulate the CSP. The other parts in the FEM were simulated by four-node bilinear plane strain quadrilateral reduced elements (CPE4R).

The contact or tie is often used to simulate the interaction relationship between soil and CSP finite element analysis. The two techniques yield very close results in the induced structural deformation and internal forces [26,29]. To ensure the convergence of the FEM, the tie was used for the interaction between the CSP and soil. The same technique was used to simulate the interaction between the soil and foundation. The CSP was modeled using beam elements with linear elastic behavior and with equivalent axial and flexural stiffness based on the corresponding plate dimensions [30]. The Mohr–Coulomb failure criterion was used for simulating the backfill and native soil. Triaxial compression tests revealed that the internal friction angles for sandy silt and gravelly soil were 24.48° and 40.43°, respectively. The cohesion values for sandy silt and gravelly sand were 1.7 kPa and 13.7 kPa, respectively. The concrete foundation was simulated by a linear elastic model. More detailed material properties used for the different components in FEMs are shown in Table 1.

The model change technique in ABAQUS was used to remove the meshed backfill soil and reactivated the backfilling layer step by step in the following steps. The residual stress generated in the soil by compaction was simulated using predefined fields, and the initial horizontal earth pressure value was 50 kPa [27]. Due to the distribution of the live load along the soil layer, the live load were reduced when the 2D FEMs were used. The ratio between the load spread distance in the longitudinal direction and the cover depth was 1:2 according to the CHBDC [6].

### 5.2. Modeling Verification

#### 5.2.1. Modeling Verification for Backfilling Process

**Figure 13:**
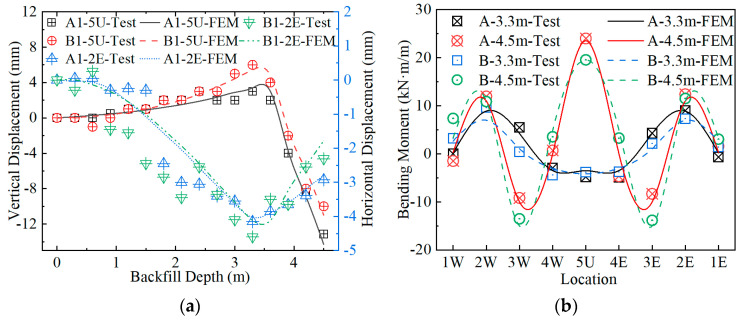
Comparisons of test data and FEM results during backfilling: (**a**) deformation; (**b**) bending moment.

The crown vertical deformation and the horizontal deformation of the middle of the sidewall during backfilling calculated by FEMs were compared with the test results, as shown in Figure 13a. It can be seen from Figure 13 that the calculated deformation trend is consistent with the measured deformation trend. When the backfilling reaches 3.3 m above the arch foot, the upper deformation of the crown is the largest, and then the deformation of the crown begins to reverse. The difference between the crown maximum upward deformation of Structures A and B calculated by the FEM and the measured values is within 13% of the test values. The difference between the crown maximum downward deformation of Structures A and B calculated by the FEM and the measured value is within 10% of the test values. The maximum horizontal deformation of the middle of the sidewall calculated by the FEM is slightly smaller than the measured value, and the error is within 11%.

The calculated bending moment of Structures A and B in the critical construction stage was compared with the measured values, as shown in Figure 13b. It can be seen from Figure 13b that the bending moment calculated by the FEM is consistent with the measured values. The calculated bending moment in the middle of the sidewall at the end of stage 2 is slightly smaller than the measured value. The haunch bending moment of Structures A and B at the end of stage 3 calculated by the FEM is slightly larger than the measured value, but the difference is within 11%. The crown bending moment is relatively close.

#### 5.2.2. Modeling Verification under Live Load

**Figure 14:**
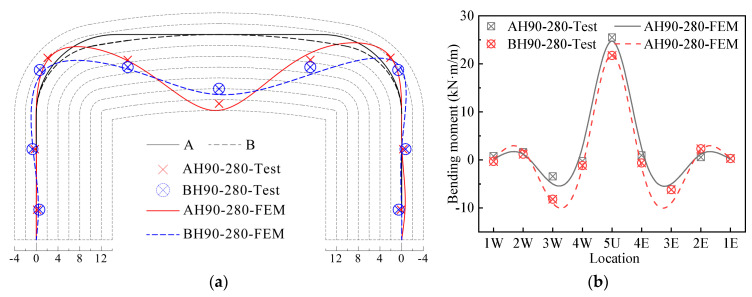
Comparisons of test data and FEM results under live load. (**a**) Displacement; (**b**) bending moment.

Taking the load of 280 kN as an example, the accuracy of the FEM under the live load was illustrated. The calculated deformation and bending moment of Structures A and B under the 280 kN live load were compared with the measured values, as shown in Figure 14a and Figure 14b, respectively. It can be seen from Figure 14a that the deformation calculated by the FEM is in good agreement with the measured deformation. The crown vertical deformation and the haunch horizontal deformation are slightly larger than the measured value, but the deformation difference in the crown is within 10%. As shown in Figure 14b, the bending moment calculated by the FEM and the measured bending moment have the same distribution, but the calculated crown bending moment is slightly smaller than the measured value, and the haunch bending moment is slightly larger than the measured value. The error of the crown bending moment is within 4%. Therefore, it can be seen from Figure 14 that the FEM in this paper can effectively predict the response of the structure under the live load.

### 5.3. Parametric Analysis

The orthogonal test is a reliable means to analyze the influence of multi-factors and multi-levels on structural performance and selects some representative points from the overall test for testing [31]. In this paper, to obtain the key factors affecting the performance of the BTSSS, the orthogonal test was used to analyze the influence of cross-section geometric parameters on the performance of the BTSSS. Generally, the span, rise, arch height, haunch radius, and inertia moment of the CSP had a significant impact on the mechanical performance of the BTSSS. Therefore, the above five different factors were selected to conduct an orthogonal test, and the above five factors were represented by A, B, C, D, and E, respectively. Four different levels were selected for each factor, denoted by 1, 2, 3, and 4, respectively. The dimensions and levels corresponding to different factors are shown in Table 2. Moreover, 16 groups of orthogonal tests formed with five factors and four levels are shown in Table 3.

**Figure 15:**
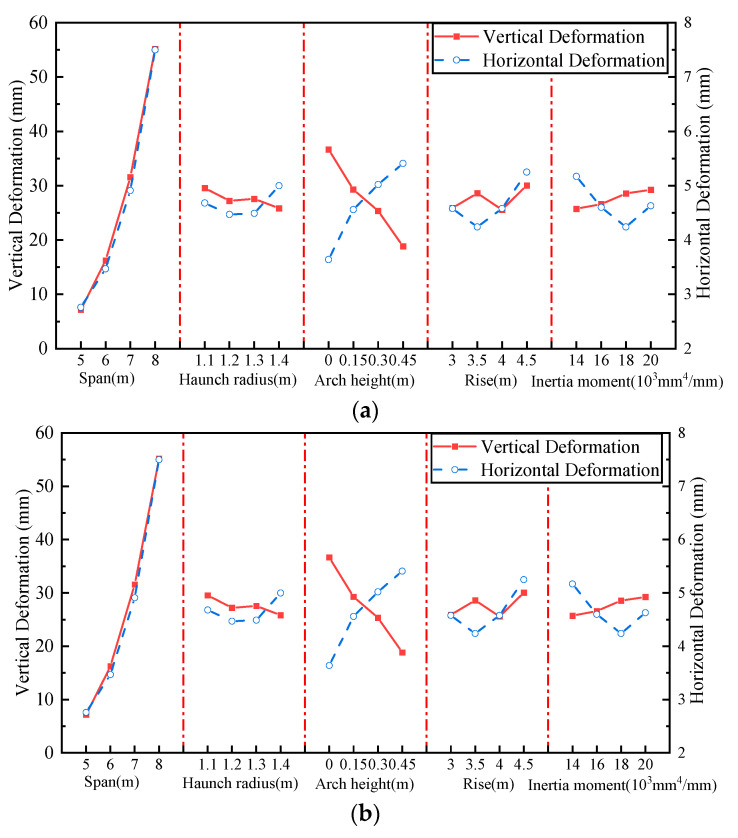
The relationship between evaluation indicators and different factors: (**a**) bending moment; (**b**) deformation.

Based on the experimental results and design codes, the crown and haunch bending moment, the crown vertical deformation, and the haunch horizontal deformation were selected as the evaluation indexes of the influence of various factors on mechanical performance. The orthogonal test results are shown in Table 3. According to the calculation results in Table 3, the relationship between the sum of the calculation results of different levels of each factor and different factors is shown in Figure 15.

It can be seen from Figure 15 that the most influential factor on the crown and haunch bending moment is the span of the structure, followed by the arch height. The bending moment and deformation increase with the increase in the span. With the increase in the arch height, the bending moment of the structure under the dead load and live load decreases linearly, and the crown vertical deformation is significantly reduced, and the horizontal deformation of the structure is significantly increased. The soil–steel interaction is exerted. The effect of span and arch height on the bending moment under the live load is more significant. The sensitivity of the bending moment to inertia moment is lower than the above two factors but significantly higher than rise and haunch radii. The increase in inertia moment makes the CSP bear more loads, and the soil–structure interaction decreases. The haunch and crown bending moments increase with the increase in the inertia moment. Rise and haunch radii have less influence on the deformation and bending moment.

## 6. Design Method Modification

It can be seen from the above analysis that the span, crown, and haunch radii have an important impact on the mechanical performance of the BTSSS. The bending moment calculation formulas of structures under live and dead loads proposed by Duncan are shown in Equations (1)–(7). It can be seen from Equations (1)–(7) that only the span in cross-section geometric parameters is considered, and the crown and haunch radii are not. Duncan’s research results show that the sum of the absolute values of the crown and haunch bending moments is directly related to the span, cover depth, and axle load. The crown and haunch bending moments is calculated based on the sum of the absolute values of the crown and haunch bending moments and the moment distribution coefficient κ.

(1) $$M_{D}=k_{1}\gamma {D_{h}}^{3}+k_{2}\gamma \left(H-0.3-d_{c}/2000\right){D_{h}}^{2}$$

(2) $$M_{L}=C_{1}k_{3}L_{L}D_{h}$$

(3) $$M_{\mathrm{cD}}=\kappa M_{D}$$

(4) $$M_{\mathrm{hD}}=\left(1-\kappa \right)M_{D}$$

(5) $$M_{\mathrm{cL}}=\kappa M_{L}$$

(6) $$M_{\mathrm{hL}}=\left(1-\kappa \right)k_{R}M_{L}$$

(7) $$\kappa =0.7-0.0328D_{h}$$

where $M_{D}$ and $M_{L}$ represent the sum of the absolute values of the crown and haunch bending moments under the dead load and load live load, respectively (kN∙m/m); $M_{\mathrm{cD}}$ and $M_{\mathrm{cL}}$ represent the crown bending moment under the dead load and live load, respectively (kN∙m/m); $M_{\mathrm{hD}}$ and $M_{\mathrm{hL}}$ represent the bending moment under the dead load and live load, respectively (kN∙m/m); $d_{c}$ is the rise in the structural plate corrugations (mm); $D_{h}$ is the span of the structure (m); γ is the bulk density of the backfill (kN/m^3^); H is the cover depth (m); $k_{1}$ and $k_{2}$ are the factors used in calculating the dead load moment, $k_{1}=0.0053-0.00024\left(3.28D_{h}-12\right)$, $k_{2}=0.053$; $C_{1}$ is the tandem axle coefficient, $C_{1}=1.0$ for single axles and $C_{1}=0.5+\frac{D_{h}}{15.24}\leq 1.0$ for multiple axles; $k_{3}$ is the factor used in calculating the live load bending moment, $k_{3}=\frac{0.08}{{\left(H/D_{h}\right)}^{0.2}}$ for $D_{h}\leq 6\;m$, $k_{3}=\frac{0.08-0.002\left(3.28D_{h}-20\right)}{{\left(H/D_{h}\right)}^{0.2}}$ for $6\;m<D_{h}<8\;m$; $L_{L}$ is the line load equivalent to the live load, $L_{L}=A_{L}/k_{4}$; $A_{L}$ is the axle load; $k_{4}$ is the factor used in calculating equivalent line loads; and κ is crown bending moment distribution coefficient.

This paper only corrects the calculation method of the sum of the absolute values of the crown and haunch bending moment under the dead load $M_{D}$. Since the surface distributed load was used to represent the axle load, the calculation method of the bending moment under the vehicle load is not modified. The bending moment distribution coefficient κ should be fitted based on the relationship between the crown and haunch bending moments under the dead load and live load and the sum of the absolute values of the crown and haunch bending moments. This article does not modify the bending moment distribution coefficient κ. It can be seen from Equation (1) that the $M_{D}$ is mainly divided into two parts. One part is the bending moment when the backfill reaches the minimum height of the cover (0.3 m), and the other part is the bending generated in backfilling the remaining part.

**Figure 16:**
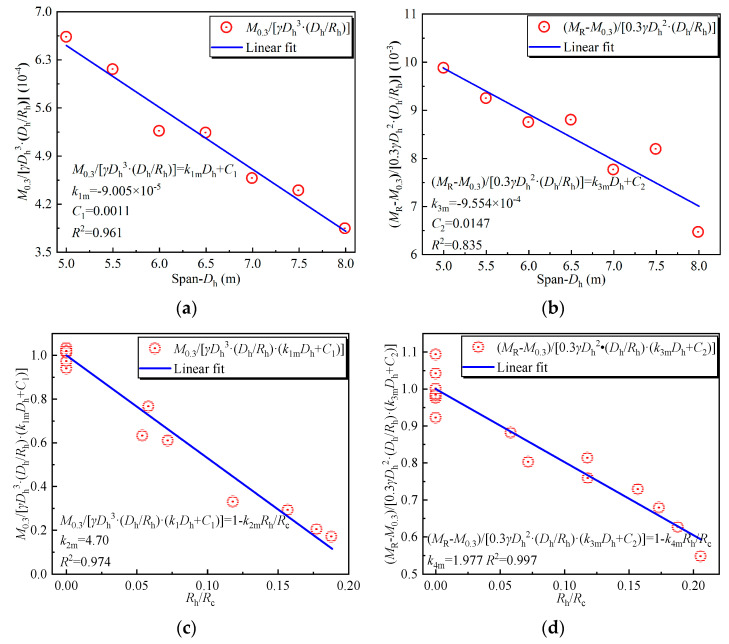
Linear fitting of parameters. (**a**) $k_{1m}$ and $C_{1}$; (**b**) $k_{3m}$ and $C_{2}$; (**c**) $k_{2m}$; (**d**) $k_{4m}$.

Factors such as the ratio of the haunch radius to the crown radius $R_{h}/R_{c}$ and the ratio of the span to the haunch radius $D_{h}/R_{h}$ were added to Equation (1), as shown in Equation (8). When the crown of the structure is straight, $1-k_{2}(R_{h}/R_{c})$ and $1-k_{4}(R_{h}/R_{c})$ in Equation (8) are 1, and the factors $k_{1m}$, $C_{1}$, $k_{3m}$, and $C_{2}$ can be firstly fitted. In order to have more samples to fit the factors, more FEMs were established based on the above parameter analysis. The relationship between the ratio of the bending moment when the backfill reaches the minimum height of the cover (0.3 m) $M_{0.3}$ to $\gamma {D_{h}}^{3}\cdot \left(D_{h}/R_{h}\right)$ and the span $D_{h}$ is shown in Figure 16a. $k_{1m}$ and $C_{1}$ obtained by linear fitting are −9.005 × 10^−5^ and 0.0011, respectively. The relationship between the ratio of the bending moment generated in backfilling the remaining part $M_{R}-M_{0.3}$ to $M\gamma {D_{h}}^{2}\left(H-0.3-d_{c}/2000\right)\left(D_{h}/R_{h}\right)$ and the span $D_{h}$ is shown in Figure 16b. $k_{3m}$ and $C_{2}$ obtained by linear fitting are −9.554 × 10^−4^ and 0.0147, respectively. After the factors $k_{1m}$, $C_{1}$, $k_{3m}$, and $C_{2}$ were fitted, the $k_{2m}$ and ${k_{4}}_{m}$ were linearly fitted as shown in Figure 16c,d. $k_{2m}$ and ${k_{4}}_{m}$ are 4.70 and 1.977, respectively. It can be seen from Figure 16 that the fitting errors of $k_{1m}$, $C_{1}$, $k_{2m}$, and $k_{4m}$ are within 5%. The fitting error of $k_{3m}$ and $C_{2}$ is slightly larger, about 16.5%.

(8) $$M_{D}=\frac{D_{h}}{R_{h}}\left(k_{1m}D_{h}+C_{1}\right)\left(1-k_{2m}\frac{R_{h}}{R_{c}}\right)\gamma {D_{h}}^{3}+\frac{D_{h}}{R_{h}}\left(k_{3m}D_{h}+C_{2}\right)\left(1-k_{4m}\frac{R_{h}}{R_{c}}\right)\left(H-0.3-\frac{d_{c}}{2000}\right)\gamma {D_{h}}^{2}$$

where $R_{h}$ is the haunch radius (m); $R_{c}$ is the crown radius (m); $k_{1m}$, $k_{2m}$, $k_{3m}$, and $k_{4m}$ are bending moment solution coefficients; and $C_{1}$ and $C_{2}$ are constants.

## 7. Conclusions

The effect of the crown and haunch radii on the mechanical performance of the BTSSS during the backfill process and under the live load was studied via full-scale model tests. The finite element models were established and used to analyze the influence of cross-section geometric parameters on the mechanical performance of the BTSSS. A more accurate formula for calculating the bending moment in the backfilling process of the BTSSS was fitted. The conclusions are listed as follows:

(1) The upward deformation and the stress in the middle of the sidewall are the largest when the backfill reaches the haunch. The BTSSS with the straight crown (Structure A) has less upward deformation than the BTSSS with the curved crown (Structure B). The haunch stress and bending moment of Structure A are significantly greater than those of Structure B.
(2) After the backfill reaches the haunch, the deformation begins to reverse. At the end of backfilling, the deflection, bending moment, and earth pressure of Structure A are 1.3 times, 1.2 times, and 2.2 times those of Structure B, respectively. Structure B promotes more even haunch and crown bending moments and earth pressures through stronger soil–steel interactions.
(3) The deformation of Structure A under the live load is greater than that of Structure B at the same cover depth. The deformation of Structure A is twice that of Structure B when the cover depth is 60 cm. The crown bending moment of Structure A is twice that of Structure B when the cover depth is 30 cm.
(4) The span and arch height of the cross-section are the most important factors, which need to be designed emphatically.
(5) Based on the CHBDC specification, a more accurate calculation formula for calculating the bending moment during the backfilling process of the BTSSS was proposed. Incorporating the crown and haunch radii into the revised calculation formula, the formula has wider applicability.

This article provides valuable insights into the design of BTSSSs. However, the design method in this article needs to be verified with more field test data. At the same time, the internal force under vehicle load was also related to the crown and haunch radii. Due to the site limitation, the vehicle load was simulated by surface distributed load in this article. Therefore, the calculation method of the internal force under the vehicle load was not modified in this article. The calculation method of internal forces under the action of vehicle load needs to be further modified through the response data of BTSSSs under vehicle load and the analysis results of the numerical modeling.

## Figures and Tables

**Table 1:** Summary of material properties in FEM.

Properties	Sandy Silt	Gravelly Soil	Native Soil	Concrete Foundation
Density (kg/m^3^)	1796	2021	1900	2400
Elastic modulus (MPa)	18.82	62.45	30	30,000
Poisson ratio	0.3	0.3	0.3	0.2
Cohesion (kPa)	1.7	13.7	1.7	-
Friction angle (°)	24.48	40.43	24.48	-
Dilatancy angle	4	10	4	-

**Table 2:** Orthogonal design of cross-section geometric parameters.

Cross-Section Geometric Parameters	Level	Factor				
		A (m)	B (m)	C (m)	D (m)	E (mm^4^/mm)
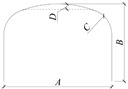	1	5	3	1.1	0.00	14,005.36
	2	6	3.5	1.2	0.15	16,072.34
	3	7	4	1.3	0.30	18,158.81
	4	8	4.5	1.4	0.45	20,265.75

**Table 3:** Test plan and calculation results.

Test Plan						Dead Load Bending Moment (kN·m/m)		Live Load Bending Moment (kN·m/m)		Deformation (mm)	
Schemes	A	B	C	D	E	Crown	Haunch	Crown	Haunch	Vertical	Horizontal
1	1	1	1	1	1	5.52	7.46	24.71	20.76	14.83	2.19
2	1	2	2	2	2	0.71	5.21	21.28	17.53	8.72	2.00
3	1	3	3	3	3	5.11	2.00	18.22	14.87	4.06	2.45
4	1	4	4	4	4	8.22	1.51	13.58	10.54	1.01	4.41
5	2	1	2	3	4	3.49	6.46	26.15	22.52	13.78	3.53
6	2	2	1	4	3	0.20	5.08	22.91	19.90	11.65	3.41
7	2	3	4	1	2	7.08	10.52	31.93	24.76	20.77	2.65
8	2	4	3	2	1	3.40	9.42	27.52	22.80	18.72	4.3
9	3	1	3	4	2	2.87	6.08	25.10	20.98	20.33	5.35
10	3	2	4	3	1	5.03	8.13	28.02	23.60	26.96	5.69
11	3	3	1	2	4	12.60	18.41	42.59	36.81	35.08	4.72
12	3	4	2	1	3	15.09	20.39	48.24	39.93	43.93	3.86
13	4	1	4	2	3	17.08	19.09	47.58	40.33	54.6	7.23
14	4	2	3	1	4	23.62	25.85	59.87	49.62	67.15	5.87
15	4	3	2	4	1	6.67	12.84	33.52	27.41	42.39	8.48
16	4	4	1	3	2	13.09	20.75	45.03	40.06	56.62	8.41

## Data Availability

The data presented in this study are available on request from the corresponding author. The data are not publicly available due to the fact that our research is continuing.
